# Impact of extrinsic factors on fine motor performance of children attending day care

**DOI:** 10.1016/j.rppede.2016.03.007

**Published:** 2016

**Authors:** Carolina Corsi, Mariana Martins dos Santos, Luísa de Andrade Perez Marques, Nelci Adriana Cicuto Ferreira Rocha

**Affiliations:** Universidade Federal de São Carlos (UFSCar), São Carlos, SP, Brazil

**Keywords:** Day care centers, Children, Preschooler, Child development, Fine motor dexterity, School health

## Abstract

**Objective:**

To assess the impact of extrinsic factors on fine motor performance of children aged 2-years old.

**Methods:**

73 children attending public and 21 private day care centers were assessed. Day care environment was evaluated using the Infant/Toddler Environment Rating Scale-Revised Edition (ITERS-R), fine motor performance was assessed through the Bayley Scales of Infant and Toddler Development-III (BSITD-III), socioeconomic data, maternal education and time of start at the day care were collected through interviews. Spearman's correlation coefficient was calculated to assess the association between the studied variables.

**Results:**

The time at the day care was positively correlated with the children's performance in some fine motor tasks of the BSITD-III, showing that the activities developed in day care centers were important for the refinement of specific motor skills, while the overall fine motor performance by the scale was associated with maternal education and the ITERS-R scale sub-item “language and understanding”.

**Conclusions:**

Extrinsic factors such as higher maternal education and quality of day care centers are associated with fine motor performance in children attending day care.

## Resumo

**Objetivo:**

Verificar as repercussões de fatores extrínsecos no desempenho motor fino de crianças de dois anos.

**Métodos:**

Foram avaliadas 73 crianças de creches públicas e 21 de creches particulares. O ambiente da creche foi avaliado com a escala Infant/Toddler Environment Rating Scale - Revised Edition (ITERS-R), o desempenho motor fino pela escala Bayley Scales of Infant and Toddler Development - III (BSITD-III), dados socioeconômicos, escolaridade materna e tempo de ingresso na creche foram colhidos por meio de entrevista*.* Foi calculado o coeficiente de correlação pelo teste de Spearmann para verificar a relação entre as variáveis avaliadas.

**Resultados:**

O tempo de creche apresentou correlação positiva com o desempenho das crianças em algumas tarefas de motricidade fina da BSITD-III, demonstrou que as atividades desenvolvidas nas creches foram importantes para o refinamento de habilidades motoras específicas, enquanto o desempenho motor fino geral pela escala estava relacionado com a escolaridade materna e com o subitem da escala ITERS-R “Linguagem e compreensão”.

**Conclusões:**

Fatores extrínsecos como maior escolaridade materna e qualidade de creches estão relacionados com o desempenho motor fino de crianças frequentadoras de creches.

## Introduction

The first years of a child's life are characterized by constant biological and psychosocial changes, which lead to major acquisitions in the motor, social-affective, and cognitive domains.^1^ During this period, the central nervous system (CNS) is constantly changing, myelination and synaptic organization reach the peak at 2 years of age, favoring the learning processes.^2^^,^ 3 However, the CNS is not the only factor responsible for motor development, it is also related to the musculoskeletal development and cardiorespiratory fitness, all influenced by stimuli and environmental factors.^4^^-^ 7 There is evidence that low socioeconomic status^8^^-^ 11 and family and school environment of poor quality can negatively influence the development of healthy children,^8^^,^ 10 ^,^ 12 ^,^ 13 while favorable environmental conditions, such as adequate stimuli, higher maternal education and socioeconomic status seem to positively influence children's motor and cognitive development.^8^^,^ 14 ^-^ 16

As for environmental factors, it is known that, since the late 1970s, women have become part of the labor market and required a place to leave their children during work hours. So, children began to spend much of their day in a different environment. However, in Brazil, with the approval of a national Law in 1996 (Lei Nacional de Diretrizes e Bases da Educação Nacional-LDB), the day care centers no longer have a social welfare aspect, but an educational character and are responsible for the “integral development of children up to 6-years old, in their physical, psychological, intellectual and social aspects, complementing the action of the family and of the community”(Art. 29 LDB). Rossetti-Ferreira et al.^17^ highlight that children insertion in day care centers offers a possibility of additional stimuli, as they interact with other children and caregivers. However, this benefit is directly related to the quality of the child care provided.

Under this new perspective, concern for the environment as a delineator factor of development in the early years of life has led some researchers to question the influence of the school environment as a space for children development,^2^^,^ 8 ^,^ 18 ^-^ 21 since the lived experiences of those years are related to the cognitive^22^^,^ 23 and motor^5^^,^ 24 development in subsequent years, many children have the day care center as their main source of stimulation and interaction. In this context, it was found that day care centers with adequate equipment, good quality in the care and teaching methodology had positive influence on children development.^2^^,^ 15 ^,^ 25 However, there are few studies assessing the school environment influence on fine motor skills of children at 2 years of age, period in which they begin to develop greater independence in daily activities and ability to use their hands functionally. Studies evaluating the fine motor performance among frequenters of Brazilian nurseries attributed the poor performance of children to the lack of quality of day care centers in Brazil.^2^^,^ 18 ^,^ 26 However, this conclusion was based on studies that evaluated only the quality of day care centers in Brazil, without relating their impact on children's performance.

In a previous study that assessed the performance of children attending public and private day care centers, all with level B of socioeconomic status and aged 0 to 3 years, children attending public day care centers showed cognitive and fine motor performance lower than those in private day care centers. These results was attributed to possible structural and pedagogical differences between the types of day care centers, as the children showed no changes in organic systems and were of the same socioeconomic level. This study, however, did not evaluate the quality of the day care environment.^27^

To the best of our knowledge, there are no studies evaluating school environment quality and assessing its influence on motor performance of children. In an attempt to fill this gap in the literature, the aim of this study was to investigate the impact of extrinsic factors, represented by the quality and length of stay in day care, level of maternal education, and family socioeconomic status, on fine motor development of children aged two years. Considering that several studies indicate the influence of environment on the development of biologically healthy children,^18^^,^ 28 our hypothesis is that the higher the level of maternal education and the family socioeconomic status and the better the quality of day care, better will be the fine motor development of children at 2 years of age. It is also expected that children early enrolled in day care centers would have a better fine motor performance, since at 2 years of age the most motivated activities at home are related to gross motor skills and, in day care centers, fine motor skills are encouraged.^27^

## Method

After surveying the Child Education Municipal Centers (CEMEIS) and private day care centers of a medium-sized city in the State of São Paulo, it was found a population of 570 children aged 2 years, 470 (83%) attending the municipal network and 100 (17%) the private schools. These children are distributed in 40 day care centers: 22 (55%) in municipal institutions and 18 (45%) in private day care centers. After sample size calculation for finite populations, with two-tailed alpha of 5% and 95% confidence interval, a sample of 80 children was estimated. This calculation does not require the definition of an outcome.

Children of both sexes, born at term (40.1±2 weeks), appropriate weight for gestational age (3.2±0.5kg), with Apgar score>7 in the first and fifth minutes of life, and within the age of two years (23±3 months) were included. At the evaluation time, all children presented with normal weight percentile and appropriate height for chronological age, according to the World Health Organization,^29^ and attending full-time day care centers for at least six months (±14.8 months). Children with neurological disorders, genetic syndromes or congenital malformations were excluded. Parents or guardians have authorized their participation and gave written informed consent. Children were excluded from the study if, during the evaluation, they were crying or showing irritation that could prevent the application of tests or any health condition that could hinder the evaluation.

After approval by the Institutional Review Board of the Centro Universitário Central Paulista (No 31/2011), all full-time day care centers of a city in the interior of São Paulo who served children aged two years were invited to participate. Among the 13 public day care centers, three were excluded due to lack of space for motor performance evaluation. Among the 28 private day care centers invited, only nine agreed to participate. There is no way of knowing whether there are significant differences between the evaluated day care centers and the ones who refused to participate, as the researchers were prevented from entering the non-participating centers. In the participating day care centers, all children aged two years were invited and evaluated all children whose parents or guardians gave WIC.

Data collection consisted of physical evaluation and application of a questionnaire to parents to gather birth data, health conditions, age enrollment in day care, and maternal education. For assessment of the family's socioeconomic status, the ABEP questionnaire (Associação Brasileira de Empresas de Pesquisa/Brazilian Market Research Association) was used for economic classification.^30^ The questionnaire uses characteristics of the home (number of TV, radio, bathroom, car, housemaid, washing machine, DVD, refrigerator, freezer, and education level of the head of the household) to quantify the socioeconomic level at A, B, C, D or E.^30^

The children were called in their rooms and sent to a place determined by the day care direction for motor performance evaluation and anthropometric measurements. It is noteworthy that the researcher observed the hours of sleep, feeding, and bathing proposed by the institutions, and if a child was crying and refused to do the activities, he/she was immediately conducted to his/her room.

The assessment tools used were the Bayley Scales of Infant and Toddler Development, Third Edition (BSID-III) and Infant/Toddler Environment Rating Scale, Revised Edition (ITERS-R).

BSITD-III is a reliable developmental rating scale, which is validated for children 0-42 months^31^ to measure cognitive, motor, language, social-emotional, and adaptive behavior. BSITD-III application occurs according to the child's age, with the first evaluation task corresponding to the age. In the present study, only the fine motor domain was evaluated. The tasks were scored by assigning 1 when the child was able to do the activity, attend certain requirements in the scale manual.^31^ The task was not scored (score 0) when the child did not perform or performed improperly the activity. When starting the test, the child was asked to perform three consecutive activities, otherwise, the researcher returned to the entry corresponding to the previous age, until the child perform correctly three consecutive activities. The assessment was stopped when the child did not perform five consecutive activities. The final score was transformed into a standard score ranging from 1 to 19 points, according to the tables contained in the manual.^31^ According to the BSITD-III, a mean of 10 and a standard deviation of 3 are considered. However, for the present study, the score is reclassified into 5 levels: below the average (<7); medium-low (7-9); medium (10); medium-high (11-13); and above average (>13). For BSITD-III application, three researchers were trained and obtained an inter-observer agreement rate of 98%.

ITERS-R was developed by Harms et al.^32^ to assess day care centers and is a valid and reliable measurement tool of physical quality and human resources in these institutions. It consists of 39 items grouped into seven subscales to evaluate the school environment: space and furnishings, personal care routines, language and literacy, learning activities, interaction, program structure, and adult needs.

Each item score varies from 1 to 7: inappropriate (1-2); minimum (3-4); good (5-7). The score for each subscale and the overall average of these scores were calculated. For scale application, three researchers were trained and obtained an inter-observer agreement rate of 96%.

For data analysis, fine motor performance was divided into five categories according to the BSITD-III manual, as mentioned above.^31^ However, we found no result below average, and four categories were assessed. Data regarding the day care center quality were also reclassified into two categories: good quality (score≥5) and poor quality (score<5). Data were processed using the SPSS Statistics 17 software (IBM Corporation, New York, United States of America). For normality test of fine motor performance, the Kolmogorov-Smirnov test was used, which indicated the non-normality of the data. Spearman's test was used to assess the relationship between fine motor development of children and extrinsic factors: quality and length of stay in day care centers, maternal education and socioeconomic status of the family. For correlation analysis, we used Dancey and Reidy classification^33^: r_s_=0.10-0.30 is a weak association; r_s_=0.40-0.60 is a moderate association; and r_s_=0.70-1 is a strong association, “s” refers to Spearman's test. A 5% significance level was adopted.

## Results

**Figure 1:**
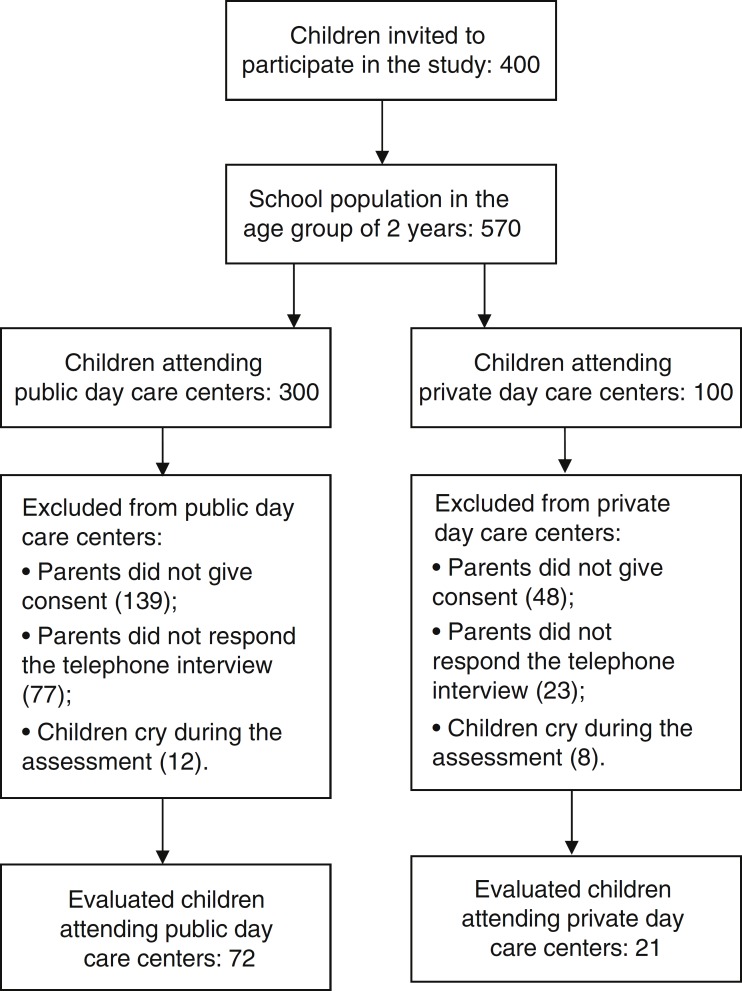
Selection criteria for participants.

Fig. 1 shows the participants selection criteria, exclusion criteria, and final sample.

Table 1 shows the fine motor performance, distribution of maternal education and socioeconomic classification of children.

**Table 1:** Frequency of participants according to level of fine motor performance (BSITD III), maternal education and socioeconomic level (Classification ABEP).

	Number of children	Frequency of children
*Fine motor*		
Medium-low	17	18%
Medium	17	18%
Medium-high	47	51%
Above average	12	13%

*Maternal education*		
Incomplete elementary school	8	9%
High school	50	54%
Higher education	19	20%

*ABEP Classification*		
C	36	38%
B	54	58%
A	4	4%

Table 2 shows the results of the correlation between fine motor performance in tasks and the BSITD-III standardized score, maternal education, ABEP classification, day care time, and the school environment characteristics. Positive and weak correlations were found between fine motor activities and day care time, maternal education and fine motor performance (r_s_=0.247; *p*=0.017), maternal education and the ITERS-R space and furniture items (r_s_=0.327; *p*=0.001), routine personal care (r_s_=0.352; *p*=0.001), language and understanding (r_s_=0.294; *p*=0.004), activities (r_s_=0.464; *p*=0.000), interaction (r_s_=0.253; *p*=0.015), program structure (r_s_=0.381; *p*=0.000), parents and staff (r_s_=0.464; *p*=0.000), and overall quality (r_s_=0.294; *p*=0.004).

**Table 2:** Correlation between fine motor performance by tasks and BSITD-III standardized score, maternal education, ABEP classification, day care time, and characteristics of the school environment.

Item	^37^			^40^			^42^			^44^			^47^			^50^			^54^			Fine motor			Maternal education	
	r_s_	*p*-value		r_s_	*p*-value		r_s_	*p*-value		r_s_	*p*-value		r_s_	*p*-value		r_s_	*p*-value		r_s_	*p*-value		r_s_	*p*-value		r_s_	*p*-value
Maternal education	0.077	0.465		0.034	0.745		0.084	0.428		0.019	0.859		0.022	0.836		0.082	0.435		0.081	0.444		0.247	0.017		-	-
ABEP classification	0.121	0.249		0.001	0.996		0.053	0.614		0.106	0.310		0.091	0.384		0.112	0.285		0.150	0.151		-0.106	0.314		0.642	**0.001**
Day care time	0.318	**0.002**		0.355	**0.001**		0.356	**0.001**		0.316	**0.002**		0.288	**0.005**		0.319	**0.002**		0.394	**0.001**		0.178	0.088		0.025	0.812
Space and furnishings	0.083	0.430		0.095	0.363		0.101	0.334		0.120	0.253		0.111	0.291		0.210	0.252		0.083	0.429		0.095	0.363		0.327	**0.001**
Personal care	0.077	0.465		0.069	0.508		0.002	0.987		0.140	0.180		0.031	0.769		0.050	0.633		0.060	0.567		0.172	0.099		0.352	**0.001**
Language and understanding	0.089	0.397		0.025	0.812		0.035	0.742		0.149	0.155		0.061	0.565		0.081	0.440		0.105	0.319		0.241	**0.020**		0.294	**0.004**
Activities	0.093	0.374		0.006	0.957		0.030	0.777		0.100	0.343		0.081	0.443		0.088	0.404		0.124	0.238		0.132	0.207		0.464	<**0.001**
Interaction	0.034	0.750		0.039	0.712		0.084	0.424		0.108	0.305		0.066	0.529		0.087	0.407		0.044	0.677		0.009	0.934		0.253	**0.015**
Program structure	0.071	0.497		0.039	0.709		0.003	0.975		0.109	0.297		0.043	0.681		0.063	0.549		0.078	0.455		0.189	0.070		0.381	<**0.001**
Parents and staff	0.093	0.374		0.006	0.957		0.030	0.777		0.100	0.343		0.081	0.443		0.088	0.404		0.124	0.238		0.132	0.207		0.464	<**0.001**
General	0.089	0.397		0.025	0.812		0.035	0.742		0.149	0.155		0.061	0.565		0.081	0.440		0.105	0.319		0.151	0.149		0.294	**0.004**

r_s_, value of Spearman's correlation; Activities: 37, use of tripod to take crayons and scrabble; 40, draw horizontal line; 42, gather shape sorting; 44, copy train blocks; 47, pinking paper; 50, copy block wall; 54, piling eight blocks.

Table 3 shows the distribution of maternal education, socioeconomic classification, and quality of day care centers at different levels of fine motor performance.

**Table 3:** Distribution of maternal education, socioeconomic classification, and quality of day care centers at different levels of fine motor performance.

Fine motor performance	Maternal education					ABEP classification				Day care quality	
	Elementary		Complete High school	Complete Higher education		A	B	C		Poor	God
	Incomplete	Complete									
Medium-low	25%	33.3%	16%	5.2%		0%	16.7%	22.9%		23.5%	4%
Medium	25%	13.4%	24%	5.3%		25%	18.5%	17.1%		17.7%	20%
Medium-high	50%	40%	46%	73.7%		75%	48.1%	51.4%		45.6%	64%
Above average	0%	13.3%	14%	15.8%		0%	16.7%	8.6%		13.2%	12%

## Discussion

In general, the results of this study have shown that factors such as maternal higher education, greater time spent in day care centers, and better quality of day care centers were related to the fine motor performance of children.

The BSITD proposed activities for this age group involve stacking blocks, drawing simple shapes, shape sorter, build three-dimensional figures with blocks, and cut paper.^31^ Children attending day care for a longer period of time had higher scores in these activities. Children stay in day care is still quite controversial in the literature. A study^11^ indicates that a more extended time in day care is harmful to children's development, particularly among children attending early care. However, Votruba-Drzaletal^34^ report that it is harmful only when there is low quality of care. The relationship found in the present study shows that although such tasks may have been stimulated at home, the extended time in day care favored the acquisition of several important skills for fine motor development and school learning.

In this study, we found a positive relationship between maternal education and the environmental quality of day care centers attended by children; it was shown that mothers with higher education choose higher quality day care for their children. Similarly, Sylva et al.^14^ reported that mothers with higher levels of education choose school environments that are richer in stimuli for child development, as they understand the day care center as an important environment for development, and not just a child-care place during their working time. Regarding the socioeconomic factor, there was no correlation with the child's performance, although it is a risk factor well described in the literature.^8^^,^ 11 ^,^ 28 It is believed that this lack of correlation is due to the small amount of children in socioeconomic class A. This prevented the representation of higher class children in this study.

There was no association between the overall quality of day care center measured using ITERS-R and fine motor development of children. The small number of children attending good quality day care may have contributed to these results, as 76% of the children attending good quality day care had above average performance, while that percentage was 58.8% in the population attending poor quality daycare. Authors who identify the school environment as a risk factor for child development reported routines with predominance of activities dedicated to nutrition and hygiene.^2^^,^ 8 ^,^ 19 ^,^ 21 However, when considering the quality items of classrooms using the ITERS-R, only the item oral language and understanding were related to fine motor performance. This item assesses the opportunity for children to express themselves in the classroom, how teachers foster children communication and how they strive to be understood. Appropriate language stimulation enables the interaction of children in the school environment, favors their stimulation and creates links between them, the teacher, and classmates, which promotes child development in general^13^^,^ 28 and appears to be linked to fine motor performance of the assessed children. Another aspect related to language is the influence of the ability of children to communicate and understand tasks during the fine motor performance assessments according to Bayley, as during the assessment they needed to understand the commands of investigators and repeat the proposed activities. Children who could better understand the proposed task could do it more easily, while others may have failed to do the activities for failing to understand what was proposed. According to the Embodiment cognition theory,^35^ children build the representation of objects through their physical interaction with them and, in this process, multiple factors interact, including brain and body, context and prior learning. Thus, it is believed that children in day care centers who received better language stimulation performed better in the evaluation because they understand and know better the proposed tasks.

It is believed that the other items assessed in day care centers have no correlation with fine motor performance due to the small number of good quality day care centers, particularly in the item related to the quality of different pedagogical activities developed in day care centers, such as music lessons and fine and gross motor activities, among others. This is due mainly to the difficulty of access to private day care centers, as few agreed to participate in the study due to the assessment time and difficulty of reconciling the activities in day care with the BSITD application.

Another important factor to highlight is that the school environment influence is often reported on cognitive development. Thus, the issues assessed with the use of ITERS-R may not have addressed important points for fine motor development of children, as the scale generally approaches aspects related to cognitive stimulation in children, such as interaction with teachers, language stimulation, and established routines, among others. Therefore, it can be considered that there is lack of instruments to evaluate the school environment focusing on motor development of children and scales that are more representative of motor performance are required.

Therefore, future studies assessing wider variety of school settings should be performed in order to clarify how to establish a relationship between fine motor performance and school environment quality.

As a study limitation, although the sample size calculation has been observed, we had a convenience sample; all children who did not cry during evaluation and whose parents agreed to participate were included. In this context, we believe that the study has a good external validity for similar conditions; that is, children attending public and private day care centers in medium-sized cities. Thus, there is a gap for future studies related to fine motor development in children attending day care centers of different quality or in settings different from the one evaluated.

Factors such as higher maternal education, extended stay in day care, and day care centers of better quality, especially regarding language stimulation, were associated with fine motor performance of children attending day care centers. Therefore, it is important that children attending public or private day care centers receive good stimulus to promote child development.
